# Detection of an *MN1::ETV6* Gene Fusion in a Case of Acute Myeloid Leukemia with Erythroid Differentiation: A Case Report and Review of the Literature

**DOI:** 10.1155/2023/9771388

**Published:** 2023-07-03

**Authors:** Lauren A. Choate, Liuyan Jiang, Mariam I. Stein, Wei Shen, Linda B. Baughn, Jess F. Peterson

**Affiliations:** ^1^Division of Laboratory Genetics and Genomics, Department of Laboratory Medicine and Pathology, Mayo Clinic, Rochester, Minnesota, USA; ^2^Department of Laboratory Medicine and Pathology, Mayo Clinic, Jacksonville, Florida, USA; ^3^Department of Biomedical Statistics and Informatics, Mayo Clinic, Rochester, Minnesota, USA; ^4^Division of Hematopathology, Department of Laboratory Medicine and Pathology, Mayo Clinic, Rochester, Minnesota, USA

## Abstract

The *MN1::ETV6* gene fusion resulting from t(12;22)(p13;q12) has been rarely reported in myeloid neoplasms. We describe a 69-year-old male with newly diagnosed acute myeloid leukemia (AML) with erythroid differentiation and t(12;22)(p13;q12) demonstrated by conventional chromosome studies. Subsequent fluorescence *in situ* hybridization studies demonstrated a balanced *ETV6* gene rearrangement (at 12p13). To further characterize this translocation, whole-genome sequencing was performed which confirmed t(12;22) with breakpoints involving the *MN1* and *ETV6* genes. Herein, we describe our case and review the literature to summarize the clinical and laboratory findings in patients with this rare but recurrent *MN1::ETV6* gene fusion observed in myeloid neoplasms. Importantly, this case expands the clinical spectrum associated with the *MN1::ETV6* gene fusion to include AML with erythroid differentiation. Lastly, this case demonstrates the importance of moving toward more comprehensive molecular testing to fully characterize the driver events in neoplastic genomes.

## 1. Introduction

The *ETV6* gene (at 12p13.2) encodes a transcription factor that plays an important role in hematopoiesis; however, when altered, it plays a role in leukemogenesis [1, 2]. Gene fusions involving the *ETV6* gene are common abnormalities in hematologic neoplasms, primarily observed in B-lymphoblastic leukemia/lymphoma (B-ALL/LBL), and more than 30 gene fusion partners have been described in the literature [3]. The *MN1* gene is a rarely reported gene fusion partner of *ETV6* and has been identified in several myeloid neoplasms, including acute myeloid leukemia (AML), accelerate phase chronic myeloid leukemia (AP-CML), and myelodysplastic syndrome (MDS) [4]. The *MN1* gene (at 22q12.1) encodes a transcription co-regulator, and overexpression has been identified as a poor prognostic indicator in AML [5]. Herein, we report an *MN1::ETV6* gene fusion observed in a case of AML with erythroid differentiation, a morphologic feature that has not been previously reported in the literature.

### 1.1. Clinical History and Hematopathologic Evaluation

A 69-year-old male with a past medical history of hypertension and diabetes presented to the emergency department with fatigue, shortness of breath, dizziness, and generalized weakness. His complete blood count demonstrated severe pancytopenia with a hemoglobin of 4.9 g/dL (reference range: 11.2–15.8 g/dL), white blood cell count of 4.2 × 10(9)/L (reference range: 3.7–12.1 × 10(9)/L), and a platelet count of 8 × 10(9)/L (reference range: 179–450 × 10(9)/L).

**Figure 1:**
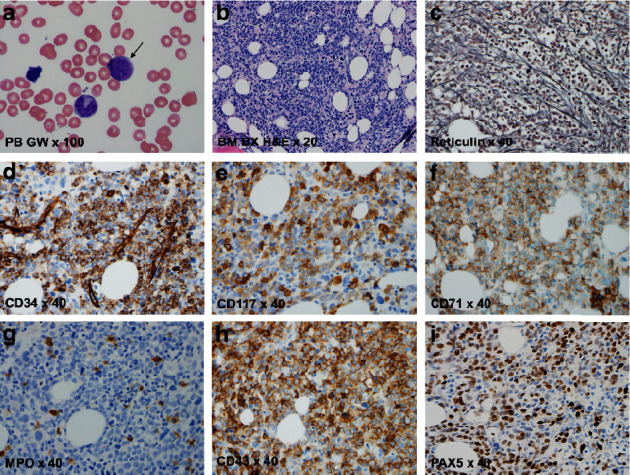
Peripheral blood and bone marrow evaluation. (a) The peripheral blood smear showed circulating blasts (arrow, Giemsa and Wright stain ×100). (b) The bone marrow biopsy was hypercellular (H & E × 20) with moderate myelofibrosis, (c) confirmed by reticulin stain (×40). (d–f) The blasts are positive for CD34, CD117, and CD71 (×40), which are suggestive for erythroid lineage. (g, h) Moreover, the blasts are negative for myeloperoxidase and positive for CD43 (×40) (i) with aberrant expression of PAX5 (×40).

A peripheral blood smear showed 40% blasts with intermediate-sized nuclei with delicately reticulated chromatin, sparse basophilic cytoplasm, increased nuclear to cytoplasmic ratios, and no Auer rods (Figure 1(a)). A bone marrow biopsy demonstrated hypercellularity (∼90%), diffuse proliferation of blasts (Figure 1(b)), and moderate myelofibrosis confirmed by reticulin stain (Figure 1(c)). Immunostains on bone marrow biopsy demonstrated that blasts were positive for CD34, CD117, CD71, and CD43 and negative for myeloperoxidase. The blasts showed aberrant expression of PAX5 (Figures 1(d)–1(i)). Flow cytometric analysis on the peripheral blood specimen identified increased blasts with a myeloid phenotype (37.4% of analyzed cells) that expressed CD15 (partial), CD33, CD34, CD38 (partial), CD45 (dim), CD56 (partial), CD117 (partial), and HLA-DR and did not express CD2, CD3, CD7, CD10, CD13, CD16, CD19, and CD64. Together, these results were consistent with AML with erythroid differentiation and moderate myelofibrosis. Cytogenetic studies were subsequently performed for further subclassification.

## 2. Materials and Methods

### 2.1. Conventional Chromosome Analysis

Cells from a peripheral blood specimen were cultured, harvested, and banded utilizing standard cytogenetic techniques according to specimen-specific protocols.

Twenty metaphases were analyzed by two qualified clinical cytogenetic technologists and interpreted by a board-certified (American Board of Medical Genetics and Genomics (ABMGG)) clinical cytogeneticist.

### 2.2. Fluorescence *In Situ* Hybridization (FISH)

An *ETV6*break-apart probe (BAP; laboratory developed test) was performed on the peripheral blood specimen. The specimen was subjected to standard FISH pretreatment, hybridization, and fluorescence microscopy according to specimen-specific protocols. One hundred total interphase nuclei were analyzed by two qualified clinical cytogenetic technologists and interpreted by an ABMGG board-certified clinical cytogeneticist.

### 2.3. Whole-Genome Sequencing (WGS)

Whole-genome sequencing was performed on the peripheral blood specimen using an Illumina NovaSeq 6000 sequencer using paired-end sequencing. Libraries were prepared using the modified NEB Ultra II (New England Biolabs, Ipswich, MA) and the Nextera Flex systems (Illumina, San Diego, CA). Reads from both libraries were combined bioinformatically and analyzed. Sequencing reads were analyzed using the DRAGEN somatic pipeline (Illumina, v3.8.4) and the GRCh38 reference genome. Small variant calling and structural variant calling were performed in tumor-only mode using the default parameters.

## 3. Results and Discussion

**Figure 2:**
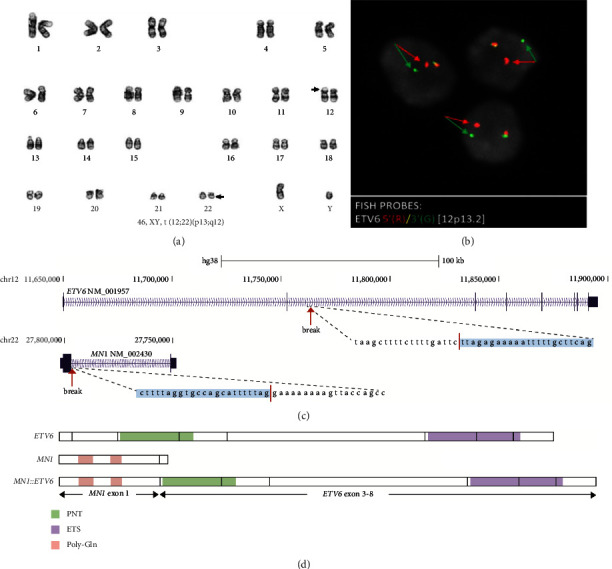
Cytogenomic evaluation of t(12;22)(p13;q12) (*MN1::ETV6* gene fusion) observed in an adult patient with AML with erythroid differentiation. (a) Representative karyogram demonstrating t(12;22)(p13;q12) (arrows). This translocation was observed in all 20 metaphases. (b) Representative interphase nuclei demonstrating a balanced *ETV6* rearrangement (break-apart probe), indicated by separated red (5′*ETV6*) and green (3′*ETV6*) signals (arrows) that flank the *ETV6* gene region (12p13). (c) Whole-genome sequencing was subsequently performed, and break-end analysis confirmed t(12;22)(p13;q12), with breakpoints located within intron 2 of *ETV6* (NM_001987.5) and intron 1 of *MN1* (NM_002430.3). (d) The resulting fusion is type I maintaining exons 3–8 of *ETV6* and exon 1 of *MN1*. PNT: pointed domain; ETS: ETS DNA-binding domain; Poly-Gln: polyglutamine domain.

Conventional chromosome analysis performed on the peripheral blood specimen demonstrated apparently balanced t(12; 22)(p13; q12) in all 20 metaphases analyzed (Figure 2(a)). The *ETV6* BAP FISH identified an apparently balanced *ETV6* gene rearrangement in 80% of 100 analyzed interphase nuclei, indicated by a single fusion signal and separated red (5′*ETV6*) and green (3′*ETV6*) probe signals (Figure 2(b)). Whole-genome sequencing was performed, and break-end analysis confirmed t(12; 22)(p13; q12), with breakpoints located within exon 2 of the *ETV6* gene (NM_001987) and exon 1 of the *MN1* gene (NM_002430) (Figure 2(c)). These breakpoints are consistent with the most common fusion type of *MN1::ETV6*, type I (Figure 2(d)) [4]. Type I fusion conserves the full HLH and ETS domains of *ETV6*, along with the majority of the *MN1* gene. In addition, no pathogenic, likely pathogenic, or variants of uncertain significance were identified from next-generation sequencing of the following genes: *CEBPA* (NM_004364.4) exon 1, *DNMT3A* (NM_022552.4) exons 8–23, *FLT3* (NM_004119.2) exons 14–20, *IDH1* (NM_005896.3) exon 4, *IDH2* (NM_002168.3) exon 4, *KIT* (NM_000222.2) exons 8–11 and 17, *KRAS* (NM_033360.3) exons 2-3, *NPM1* (NM_002520.6) exons 9–11, to −30 before exon 11, *NRAS* (NM_002524.4) exons 2 and 3, *RUNX1* (NM_001001890.2) exons 1–6, intron 4 c.725-13T>A and intron 5 c.886+1-4del, and *TP53* (NM_000546.4) exons 4–9.

Translocations involving the ETV6 gene region (at 12p13.2) are considered a frequently occurring abnormality in hematologic neoplasms, primarily observed in B-ALL/LBL [6]. The most well-described recurrent gene fusion partners of ETV6 include RUNX1 (observed in B-ALL/LBL) and PDGFRB (observed in myeloid/lymphoid neoplasms with eosinophilia) [7]. ETV6 gene alterations affect the progression of leukemogenesis through a variety of functions including constitution activation, modification of transcription factor function, loss of function, activation of proto-oncogenes, and dominant negative effects [1]. Importantly, some gene fusion partners of *ETV6* are receptor tyrosine kinases [3]. Therefore, the identification of the *ETV6* gene fusion partners is important for determining treatment options, particularly in cases with tyrosine kinase partners [8]. Interestingly, in a large study involving a cohort of approximately 10,000 patients, *ETV6* gene rearrangements were only identified in 0.5% of myeloid neoplasms, including only 1.1% of the cohort diagnosed with AML [9].

To date, the *MN1::ETV6* gene fusion has only been confirmed in 19 cases of myeloid neoplasms [4, 10–16]. The *MN1::ETV6* fusion is imprecise as some cases lack fusion transcripts and/or have breakpoints outside of either *MN1*, *ETV6*, or both [4]. Interestingly, *MN1* was found to be overexpressed in a number of AML subtypes and was also found to be overexpressed in t(12;22) cases without a confirmed *MN1::ETV6* fusion but not in *MN1::ETV6*-positive cases [4, 15]. Of cases with confirmed fusions, the most common type is type I, which preserves the super-enhancer cluster in exon 3 of *ETV6* [4]. While the exact mechanism of pathogenesis of *MN1::ETV6* gene fusions remains unclear, haploinsufficiency of ETV6 and ectopic expression of *MN1* through the *ETV6*super-enhancer may both play roles [4].

Expanding the hematopathologic spectrum of AML that harbors the *MN1::ETV6* gene fusion, our case demonstrated erythroid differentiation, which has not been previously reported in the literature. Since the bone marrow biopsy was a dry tap and no aspiration was obtained, a thorough differential count for the percentage of blasts and erythroid precursors could not be performed. Hence, the diagnostic criteria for acute erythroid leukemia (AEL) (≥80% erythroid predominance in bone marrow and ≥30% proerythroblasts) are not exactly applicable in this case [17]. Nevertheless, it has been suggested that cases with <80% erythroid predominance share similar clinicopathologic features of AEL [17]. The immunophenotype of our case shares some similarities to those of a previously reported cohort of AEL cases, including CD45, CD71, CD34, CD117, HLA-DR, and negative for myeloperoxidase. However, the common genetic features of AEL (complex or monosomal karyotypes) were not found in our case [18].

To better understand the clinical and hematopathological spectrum of myeloid neoplasms with *MN1::ETV6* gene fusions, we also summarize the previously reported cases, including our case (Table 1). The average age at diagnosis was 38.6 years old (*n* = 19), with a range of 3–69 years. The male to female ratio was 1.7 : 1 (*n* = 12 males, *n* = 7 females). Acute myeloid leukemia was the most common diagnosis, present in twelve patients, including one case of therapy-related AML. However, no erythroid differentiation was described in these AML cases. Mixed-phenotype acute leukemia (T/myeloid), chronic myelomonocytic leukemia, and MDS were reported in two patients each. Lastly, AP-CML was the diagnosis in one patient. Six patients had t(12;22) as the sole cytogenetic abnormality. Of those patients with additional chromosomal abnormalities, the most common abnormality was trisomy 8, present in nine patients. RT-PCR was the most common testing strategy to confirm the *MN1::ETV6* gene fusion (*n* = 16). Unbiased testing strategies, such as RNA fusion testing and WGS, were also performed in two cases.

As we continue to identify clinically significant gene fusions, cytogenomic laboratories need to adapt technologies that enable their detection without FISH. It is unpractical to validate and perform FISH testing for every potential gene fusion. Alternatively, next-generation sequencing technologies enable whole-genome evaluation of neoplasms. A recent study found that the of myeloid neoplasms was able to detect all abnormalities detected by conventional chromosome studies and additional findings in approximately 25% of the cases [19]. Their application as a method to detect gene fusions in an unbiased approach, either through WGS or RNA-Seq, ensures that rare fusions, such as* MN1::ETV6*, can be fully characterized and that patients can receive a more complete risk assessment.

## 4. Patient Outcome

The patient received 3 + 7 induction chemotherapy. However, a bone marrow biopsy following treatment demonstrated increased blasts (positive for CD34, CD117, and CD71) in large aggregates, demonstrating residual disease. The patient next received 2 + 5 re-induction chemotherapy. A follow-up bone marrow biopsy revealed increased blasts present in 50% of the marrow, indicating persistence of disease. The patient received three cycles of salvage therapy with decitabine plus venetoclax which resulted in complete response with blasts <5%. Currently, the patient is expected to receive an allogenic hematopoietic stem cell transplant.

## Figures and Tables

**Table 1:** Summary of *MN1::ETV6*-positive cases observed in myeloid neoplasms.

Reference	Age (years)	Sex	Diagnosis	Karyotype	Confirmatory method
Our case	69	M	AML	46, XY, t(12; 22)(p13; q12)	WGS
Buijs et al. [10]	19	F	AML-M4	47, XX, + 8, t(12; 22)(p13; q12)	RT-PCR, sequencing
Chen et al. [11]	53	F	AML-M0	47, XY, + 9, t(12; 22)(p13; q12)[9]/46, XY[l]	RT-PCR
Nakazato et al. [12]	63	M	AML-M2	46, XY, t(12; 22)(p13; q12)[20]	RT-PCR, sequencing
Rosenzweig et al. [14]	3	M	AML-DS	47, XY, + 8, + 21c, t(12; 21; 22)(p13; q22; q12)	RNA-Seq
Shao et al. [15]	24	M	AML-M0	45, X, − Y, t(12; 22)(p13; q12)[10]	RT-PCR, sequencing
Shao et al. [15]	36	M	AML-M0	46, XY, t(12; 22)(p13; q12) [7]/46, XY[3]	NA
Shao et al. [15]	62	F	AML-M4	47, XX, + 8, t(12; 22)(p13; q12)[10]	RT-PCR
Shao et al. [15]	21	F	AML-M4	48, XX, + 8, t(12; 22)(p13; q12), + 22[20]	RT-PCR, sequencing
Wang et al. [4]	46	M	AML-M5	49, XY, + 8, t(12; 22)(p13; q12), + 21, + der(22), t(12; 22) (p13; q13)[20]/50, idem, + 18[1]	RT-PCR
Wang et al. [4]	4	M	AML-M5	47, XY, + 8, t(12; 22)(p13; q12)[1]/47, idem, der(1)t(1; 13) (p32; q12), add(2)(p21), add(9)(q13), add(13) (q12)[1]/46, idem, der(1)t(1; 13)(p32; q12), add(2) (p21), − 8, add(9)(q13), add(13)(q12) [18]/46, XY[1]	RT-PCR
Nofrini et al. [13]	58	F	t-AML	47, XX, del(5)(q13q33), + 21[3]/46, idem, − 7, t(12; 22)(p13; q12)[9]	RT-PCR, sequencing
Wang et al. [4]	19	M	MPAL (T/myeloid)	47, XY, + 8, t(12; 22)(p13; q12)[14]/46, XY[6]	RT-PCR
Wang et al. [4]	11	M	MPAL (T/myeloid)	47, X, add(Y)(p11.2), t(4; 7)(q31.3; q36), + 8, t(12; 22) (p13; q12)[17]/47, idem, del(11)(q23)[2]/47, idem, t(10; 13) (p11.2; q14)[1]	RT-PCR
Wang et al. [4]	60	M	CMML	47, XY, + 8, t(12; 22)(p13; q12), del(20)(q11.2)[20]	RT-PCR
Shao et al. [15]	49	F	CMML	46, XX, t(12; 22)(p13; q12)[10]	RT-PCR, sequencing
Buijs et al. [10]	15	M	MDS	46, XY, t(12; 22)(p13; q12)	RT-PCR, sequencing
Vieira et al. [16]	63	F	t-MDS	46, XX, t(12; 22)(p13; q12)[31]/46, idem, del(20) (q11q13)[9]	RT-PCR
Buijs et al. [10]	58	M	AP-CML	46, XY, t(12; 22)(p13; q12)	RT-PCR, sequencing

M, male; F, female; AML, acute myeloid leukemia; DS, Down syndrome; t-AML, therapy-related acute myeloid leukemia; MPAL, mixed-phenotype acute leukemia; CMML, chronic myelomonocytic leukemia; MDS, myelodysplastic syndrome; t-MDS, therapy-related myelodysplastic syndrome; AP-CML, accelerated-phase chronic myeloid leukemia; WGS, whole-genome sequencing; RT-PCR, real-time polymerase chain reaction; RNA-Seq, RNA sequencing. Age, sex, diagnosis, karyotype, and MN1::ETV6 confirmation method for myeloid neoplasms harboring MN1::ETV6 fusion.

## Data Availability

The data used to support the findings of this study are available from the corresponding author upon request.
